# The Effects of Juvenile Stress on Anxiety, Cognitive Bias and Decision Making in Adulthood: A Rat Model

**DOI:** 10.1371/journal.pone.0048143

**Published:** 2012-10-31

**Authors:** Nichola M. Brydges, Lynsey Hall, Rachael Nicolson, Megan C. Holmes, Jeremy Hall

**Affiliations:** 1 Centre for Cardiovascular Science, The Queen's Medical Research Institute, University of Edinburgh, Edinburgh, United Kingdom; 2 Division for Psychiatry, Centre for Clinical Brain Science, University of Edinburgh, Edinburgh, United Kingdom; University of Queensland, Australia

## Abstract

Stress experienced in childhood is associated with an increased risk of developing psychiatric disorders in adulthood. These disorders are particularly characterized by disturbances to emotional and cognitive processes, which are not currently fully modeled in animals. Assays of cognitive bias have recently been used with animals to give an indication of their emotional/cognitive state. We used a cognitive bias test, alongside a traditional measure of anxiety (elevated plus maze), to investigate the effects of juvenile stress (JS) on adulthood behaviour using a rodent model. During the cognitive bias test, animals were trained to discriminate between two reward bowls based on a stimulus (rough/smooth sandpaper) encountered before they reached the bowls. One stimulus (e.g. rough) was associated with a lower value reward than the other (e.g. smooth). Once rats were trained, their cognitive bias was explored through the presentation of an ambiguous stimulus (intermediate grade sandpaper): a rat was classed as optimistic if it chose the bowl ordinarily associated with the high value reward. JS animals were lighter than controls, exhibited increased anxiety-like behaviour in the elevated plus maze and were more optimistic in the cognitive bias test. This increased optimism may represent an optimal foraging strategy for these underweight animals. JS animals were also faster than controls to make a decision when presented with an ambiguous stimulus, suggesting altered decision making. These results demonstrate that stress in the juvenile phase can increase anxiety-like behaviour and alter cognitive bias and decision making in adulthood in a rat model.

## Introduction

Exposure to stressful events early in life increases the risk of developing neuropsychiatric disorders later in life [1], [2], [3], [4]. Early life stressors can take a variety of forms and may be experienced in different phases of life (prenatal, early postnatal or juvenile). There is a wealth of information on the effects of stress in the perinatal phase, but comparatively little research on the juvenile phase (the childhood or pre-pubertal phase). The juvenile brain is predicted to be very sensitive to stress, as it is a ‘brain in transition’, undergoing dramatic changes in structure and function as it matures into an adult brain [5]. Research to date suggests that stress experienced in this phase is of great importance, as it is associated with the development of disorders such as depression, anxiety and PTSD, as well as impulse control disorders and suicide attempts later in life [6], [7], [8]. In animal models, juvenile stress causes lasting changes in the adult animal, increasing anxiety behaviour and altering fear conditioning, learning and memory [9], [10], [11], [12], neural gene expression (e.g. L1 and GABAa receptors [13], [14]), and increasing basal corticosterone levels and reducing neurogenesis in females only [15]. Stress in the juvenile phase also affects the animals as juveniles, remodeling cortical areas involved in emotional-type behaviours [16]. Whilst effects on behaviour are observed when animals are given stress in adulthood, they are significantly exacerbated when stress is given in juvenility [9], [17], indicating that certain changes observed in adulthood are specific to stress in this phase. These findings largely reflect what has been found in human populations, making this a suitable model for human pathologies.

Abnormal mental processing is a central component of human psychiatric disorders, resulting in disturbances in emotional and other affective behaviours. Current behavioural measurements in animal models are relatively simplistic (e.g. elevated plus maze and open field, measures of anxiety-type behaviour that can only be administered once per animal), and are not able to provide information on more complex, subjective aspects of psychiatric illness. Investigating the subjective nature of symptoms is challenging, but progress in this area would greatly enhance the translational aspects of animal models. One way of assessing affective/emotional symptoms in humans is through cognitive bias [18], [19]. The decisions an individual makes in uncertain or ambiguous situations can be influenced by their affective state, producing cognitive biases. Hence cognitive bias demonstrates a close relationship between cognitive processes and emotional state. Cognitive biases in the interpretation of ambiguous information are particularly apparent in anxious and depressed populations, who tend to interpret the emotional valence of ambiguous statements (“That is an interesting pair of shoes you are wearing”), the meaning of ambiguous homophones (e.g. die/dye, pain/pane) and the interpretation of scrambled sentences (‘winner born I am a loser)” – positive interpretation “I am a born winner”, negative “I am a born loser”) in a more negative manner than controls [20], [21]. The link between cognition and emotion is bi-directional, and cognitive biases can be used to predict emotional state [22], [23].

In recent years, assays of cognitive bias have been modified for use in non-human animals, with the aim of using their cognitive biases to inform us of their emotional state [24], [25], [26]. Studies so far have revealed that manipulations associated with negative affect (e.g. unstable housing, removal of environmental enrichment) result in animals interpreting ambiguous stimuli in a negative manner (rats [27], [28], starlings [29], honeybees [30]), and those associated with positive affect (e.g. addition of enrichment) result in positive cognitive biases [31]. Furthermore, negative cognitive biases have been observed in congenitally helpless rats (a genetic model of depression [32]), dogs with separation anxiety [33], and acutely (within hours of testing) induced in rats and chicks through the administration of pharmacological and environmental stressors [32], [34]. Interestingly, the effects of short-term environmental stress on cognitive bias were successfully pharmacologically reversed with the antidepressant imipramine in the chick model [35]. There is therefore a growing body of evidence to support the utility of cognitive bias measurements in assessing state and trait affect in animals.

In human populations, stress in early life is correlated with increased susceptibility to affective disorders and is associated with cognitive biases in adulthood [19], [36]. To date, the effects of early life stress on cognitive biases have not been studied in animal models. Successful implementation of such an assay would enhance the translational aspects of early life stress models, and may provide us with novel therapeutic targets. We therefore investigated how stress experienced in the juvenile phase affected cognitive bias in a rat model, alongside a more traditional measure of anxiety-type behaviour (elevated plus maze). We used male and female rats, as there is evidence for sex-based differences in the development of neuropsychiatric disorders in humans and animal models of perinatal stress [16], [37], [38], [39]. We predicted that animals experiencing juvenile stress would display greater anxiety-type behaviour and react more pessimistically (demonstrating a negative cognitive bias) when compared to control animals.

## Materials and Methods

### Subjects

Subjects were 24 male and 24 female Lister Hooded rats, bred from 11 adult pairs (Charles River, UK) at the University of Edinburgh. After weaning (post natal day (PND) 21), animals were housed in standard, same-sex, same-litter cages (61 cm ×43.5 cm, 21.5 cm high, Techniplast, UK), lined with wood shavings (Lillico, UK), on a 12∶12 h light/dark cycle with food (standard rat chow, RM1, Special Services Diet, Lillico, UK) and water ad libitum. Temperature and humidity were maintained between 19 and 21°C and 45 and 60% respectively. Six of the litters were assigned at random to the juvenile stress group, the remaining 5 were controls. Rats were identified via rings of permanent marker around the tail, and killed via rising concentration of CO_2_ at the end of the experiment. Rats were weighed once a week, and all procedures were carried out in strict accordance with local ethics guidelines, the UK Home Office Animals (Scientific Procedures) Act, 1986 and under a personal Home Office license (PIL number 60/10185, PPL number 60/3915).

### Juvenile Stress

The protocol used was a modified version of that presented in [14]. Rats were exposed to variable short-term stressors in the juvenile phase, on PND 25, 26 and 27, in a designated experimental room away from the regular housing area. On PND 25, animals were given a swim stress: they were placed into an opaque swim tank (25 cm high, 34 cm diameter, 12 liter capacity filled with 6 liters of water), water temperature 25+/−1°C for 10 minutes. On PND 26, animals were given restraint stress: they were placed into a plastic restraint tube (15 cm length, 5 cm diameter) for 3 periods of 30 minutes, separated by 30 minute breaks. On PND 27, animals experienced mild electric footshocks: they were given 6×0.5 mA, 0.5 s footshocks over 3 minutes (one every 30 seconds) in a rat shock chamber (30 cm ×25 cm, 32 cm high, 16 shock bars, Coulbourn Instruments, PA).

### Subjects for Elevated Plus Maze

Animals were taken from all litters for testing in the elevated plus maze (2–3 per sex per litter). Animals were housed 2–3 per cage, separated from the rest of the litter at least one week prior to testing. 12 control and 12 juvenile stressed animals were tested, aged 83 days ±11.46 S.D. at the start of testing (early adulthood). All testing was conducted blind to group.

### Elevated Plus Maze

The elevated plus maze was raised 80 cm above the floor, made of wood and painted dark grey, and comprised two open and opposite arms (70 cm ×12 cm) and two closed and opposite arms (70 cm ×12 cm and 17 cm high walls) arranged in a cross shape. A central square connected the arms (10 cm ×10 cm). During a test, an animal was placed on the central square facing an open arm. Behaviour was recorded over 5 minutes via a video recorder mounted above the maze, and tracking software (“Tracker” University of Edinburgh) was used to calculate the amount of time the animal spent in each arm and the centre of the maze. The apparatus was wiped clean between animals. The amount of time animals spent in the open vs. the closed arms (minus time spent on the central square) of the maze was compared. Animals are presumed to be more anxious if they spend a greater proportion of time in the closed compared to the open arms.

### Subjects for Cognitive Bias Test

The remaining animals (12 control, 12 juvenile stress, 2–3 per litter) were tested in the cognitive bias assay. Animals were housed 2 per cage, separated from the rest of the litter at least one week prior to testing, and were aged 99 days ±12.7 S.D. at the start of testing (early adulthood). A sample size of n = 6 per group was sufficient to detect effects in a previous study using this cognitive bias assay [31]. All testing was conducted blind to group.

### Cognitive Bias Apparatus

**Figure 1:**
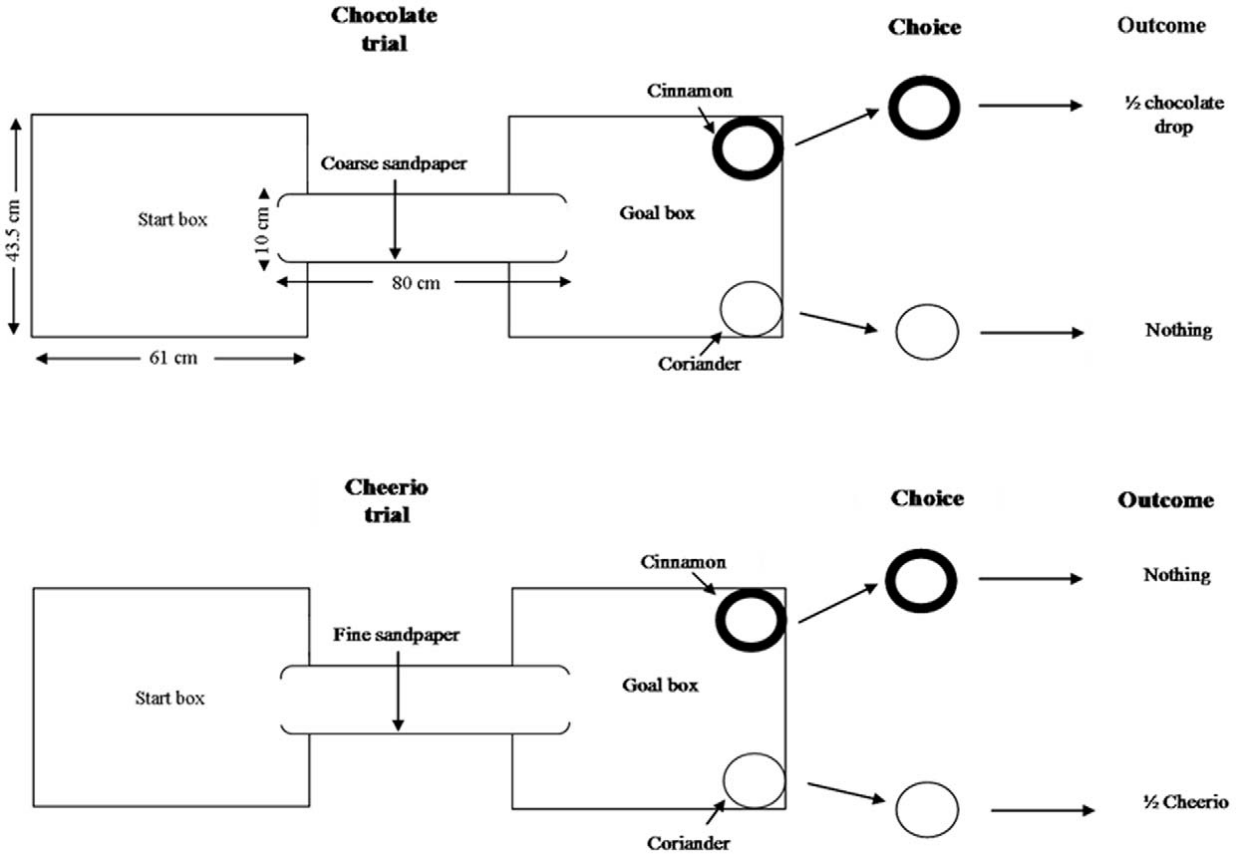
Diagram of maze apparatus with details of choice outcomes in the task. Depicted is an example where the coarse sandpaper is associated with chocolate in a black cinnamon scented bowl, and fine sandpaper with Cheerio in a white coriander scented bowl.

The maze apparatus consisted of a clear Perspex start box (61 cm ×43.5 cm, 21.5 cm high) connected to a clear Perspex goal box (61 cm ×43.5 cm, 21.5 cm high) via a piece of white Perspex drainpipe (diameter 10 cm, length 80 cm). Two foraging bowls (diameter 9 cm, height 5 cm), one black and one white, one placed on the left and one on the right, were put into the goal box (Figure 1, reproduced from [31]). This apparatus was set up in a designated testing room separate to the housing area, on a bench side (1 m high) with regular room lighting.

### Cognitive Bias Behavioural Testing

The protocol is described in full in [31], and outlined below.

### Habituation

Rats were handled daily for 10 mins each and hand fed the items to be used as rewards in the task (white chocolate drops (chocolate) and Honey Nut Cheerios (Cheerios)) for 5 days. Over the following 5 days, rats were also placed into the maze for 5 mins per day. During this stage, the maze contained the foraging bowls filled with scented sand (either coriander or cinnamon scented, 1% by weight). Each rat had a specific reward paired with a particular bowl colour, sand scent and spatial location (left or right of goal box). This remained consistent for each rat throughout the experiment (e.g. Cheerio reward always in white cinnamon bowl on left, chocolate in black coriander bowl on right), and was randomized between individuals. Pairings were counterbalanced between groups and sexes, but due to the number of possible stimulus combinations, a fully factorial design was not possible. In order to habituate the animals to the presence of sandpaper, during this stage the tunnel connecting the start and goal boxes was lined with Silicon Carbide Waterproof sandpaper (3 M, U.K.; P600 grade), which was different to sandpaper used in later stages of the experiment. In order to facilitate maximal contact (whiskers and feet) between the animal and the sandpaper, the tunnel was completely lined inside.

**Figure 2:**
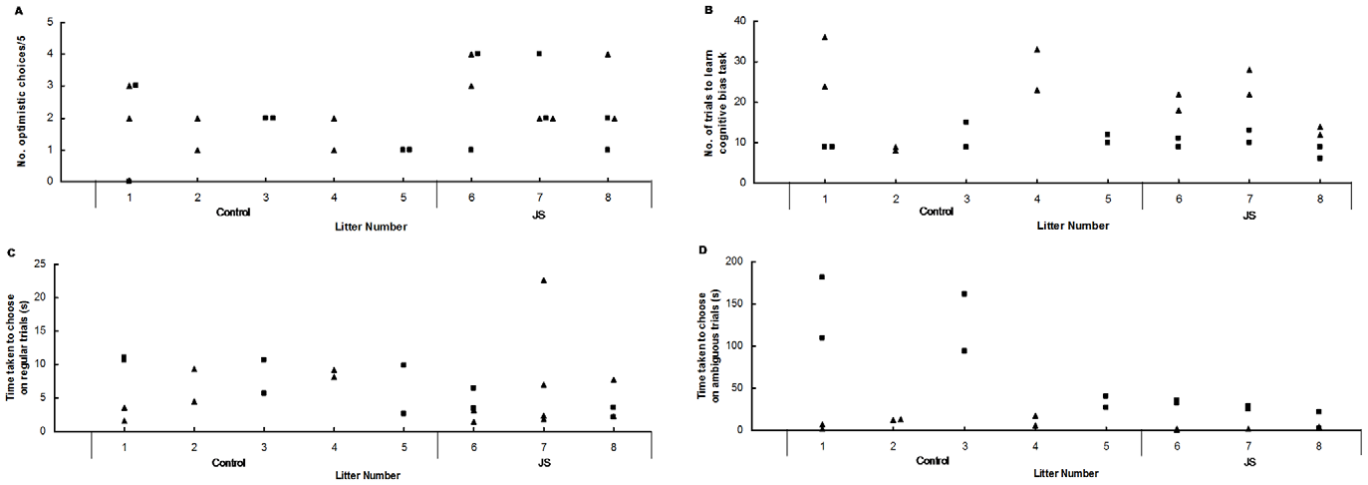
Data by litter for the cognitive bias task. A) Number of optimistic choices out of 5, B) Number of trials to learn the cognitive bias task, C) Average time taken for animals to choose a bowl in regular trials and D) Average time taken for animals to choose a bowl in ambiguous probe trials. Control litters are 1–5, JS 6–8. σ =  males, ▪  =  females.

### Pre-training

After habituation, rats were given 5 days of pre-training. They had four trials per day in the maze apparatus, two between 0900 and midday (am trials) and two between 1300 and 1700 hours (pm trials). During a trial, a reward of half a chocolate or Cheerio was put onto the surface of the sand of the appropriate bowl. Cheerios were a low value reward, chocolate high value. As both of these rewards are sweet, and it well known that many mammals, including rats, have a preference for sweet foods [40], it was assumed the rats would forage for them. Rats are expected to value the chocolate more highly than the Cheerio for several reasons: they have a higher sugar content and calorific value (0.4 kcal and 0.07 g sugar in half a Cheerio vs. 3 kcal and 0.34 g sugar per half chocolate drop), they were observed to habituate faster to eating chocolate during the habituation phase (hand feeding), and during all of the reward trials they located chocolate faster than Cheerios (see Results and previous findings in [31]). At the start of a trial rats were placed individually into the start box. A timer was started, and the time for the rat to enter and exit the tunnel, choose a bowl (which bowl was chosen, one containing reward or not) and choose the bowl containing the reward (if not chosen first) was recorded. In this phase, a choice was determined by the rat putting its face into a bowl. A rat received two trials for Cheerios and two for chocolate each day. The order of trials for a rat each day was determined using random numbers, and altered daily. Sandpaper of different grades was used to line the tunnel in this phase. Half of the rats from each group and sex had coarse sandpaper (grade P60, average particulate size 269 μm,) associated with the chocolate reward and fine sandpaper (grade P1200, average particulate size 15 μm; Faithfull Tools, Dartford, Kent, U.K.) associated with the Cheerio reward, the other half the reverse. Rats then learned to associate a particular sandpaper grade in the tunnel with a particular reward outcome, and were able to eventually choose the rewarded bowl first when entering the goal box (e.g. a rat might learn the coarse sandpaper indicated a chocolate reward in the white, cinnamon scented bowl on the left, whereas fine sandpaper meant a Cheerio in the black, coriander scented bowl on the right). The apparatus was cleaned between trials.

**Figure 3:**
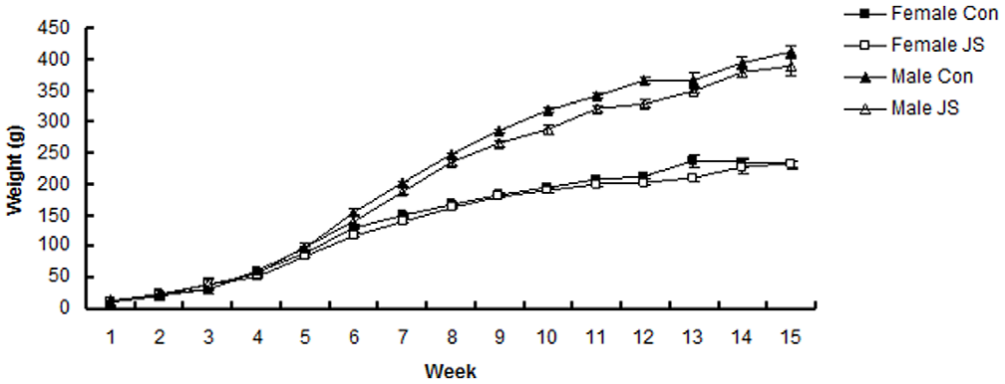
Weights of Control (Con) and Juvenile Stress (JS) male and female rats from birth to 15 weeks. Error bars represent 1 SE.

Over the next five days, the same protocol was followed, except the rewards were gradually buried further into the sand, until they were at the bottom of the bowl by day five. During this stage, choice was determined when a rat began digging in a particular bowl. Burying the rewards removes visual and olfactory reward cues, ensuring that rats are learning to associate the sandpaper with the reward outcome.

### Training

Animals were then moved onto the training stage. Here, the rewards were always buried at the bottom of the bowls. The same protocol as the previous stage was followed, with the exception that one randomly selected trial a day was not rewarded. Correct performance on these trials ensured that rats were not using any cues directly associated with the reward (e.g. olfaction). Rats did not appear to be using reward-related cues, as performance was not affected by presence or absence of reward (see Results). Once rats had completed at least three out of four trials correctly for five days in a row, we assumed they had learned the task, and they were moved onto the next stage. Therefore the duration of this phase was dependent on individual learning.

**Figure 4:**
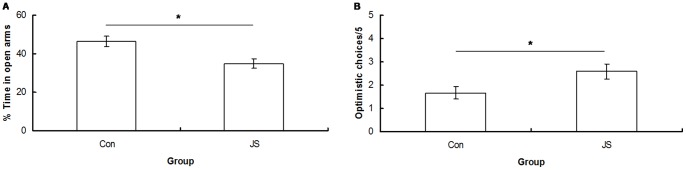
Elevated plus maze and optimistic choices in the cognitive bias test. A) % of time spent in the open arms of the elevated plus maze by Control (Con) and Juvenile stress (JS) animals. B) Mean number of optimistic choices during 5 ambiguous probe trials for Control (Con) and Juvenile Stress (JS) animals. Error bars represent 1 SE, bars connected by an asterisk are significantly different to one another.

### Probe phase

The next stage was the probe phase, and lasted for five days. In this phase, trials proceed as before, with the alteration that on randomly selected unrewarded “ blank” trials, an intermediate, ambiguous grade of sandpaper, intermediate in texture to the two training textures (P180, average particulate size 82 μm), was used instead of the sandpaper usually associated with the reward. During these trials, if rats selected the bowl usually associated with the chocolate reward this was recorded as an optimistic choice, if they chose the bowl usually associated with the Cheerio it was recorded as a pessimistic choice.

**Figure 5:**
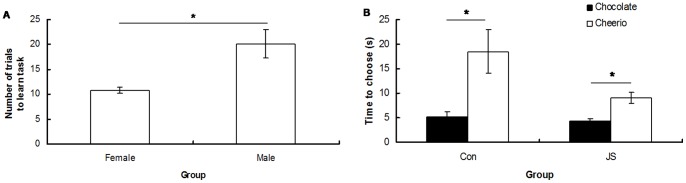
Learning rate and effect of reward type on time to choose a bowl during the cognitive bias task. A) Number of trials taken for male and female rats to learn the cognitive bias task and B) time taken for Control (Con) and Juvenile Stress (JS) animals to make a choice on chocolate vs. Cheerio trials. Error bars represent 1 SE, bars connected by an asterisk are significantly different to one another.

### Data Analysis

Unless otherwise stated, data were analysed using mixed effects ANOVA's (JMP statistical software, SAS Institute, Cary, NC, USA). All data were checked for homogeneity of variance and normality of distribution. Where these assumptions were violated, data were transformed to provide the best approximation to a normal distribution [41]. Independent variables with no measurable effect on the dependent variable (defined as p>0.1) were removed from models in a stepwise manner. The main effects are provided in the text. Where the underlying assumption of homogeneity associated with the covariance matrix was violated (e.g. in repeated measures models), degrees of freedom were automatically adjusted and may be decimal.

**Figure 6:**
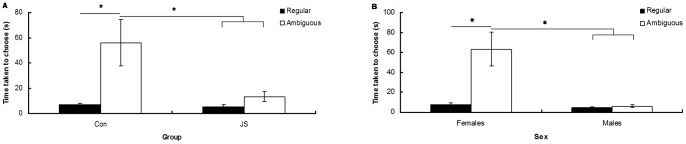
Effect of trial type on time to choose a bowl during the cognitive bias task. Average time taken to choose a bowl in regular vs. ambiguous probe trials for A) Control (Con) and Juvenile Stress (JS) groups and B) males vs. females. Error bars represent 1 SE, bars connected by an asterisk are significantly different to one another.

The first model investigated the effect of group (Control vs. JS), sex, age, sex*group, sex*age, group*age and group*age*sex interactions on weight, with animal (nested within group) fitted as a random variable. The second model investigated the effects of group, sex, litter and all two and three-way interactions of these terms on % of time rats spent in the open arms of the elevated plus maze over five minutes. A third model investigated the effect of group, sex and group*sex on number of optimistic choices in the probe phase (ambiguous trials). A chi-squared test was also conducted within groups to determine if rats were making more optimistic choices than expected by chance during the ambiguous trials. A model was set up to investigate the effect of group, sex and group*sex interaction on number of trials taken to learn the cognitive bias test in the training stage. Another model explored the effect of group, sex, trial type (chocolate, cheerio or blank), group*sex, group* trial type, sex* trial type and group*sex*trial type on correct selection of rewarded bowl, with animal (nested in group) added as a random factor. A further model investigated the effect of reward (chocolate vs. cheerio), sex, group, sex*group, sex*reward, reward*group and reward*group*sex interactions on time taken to make a choice in training trials. A final model investigated the effects of group, trial type (regular vs. intermediate, ambiguous trial), sex, group*sex, trial type*sex, trial type*group and trial type*group*sex interactions on time taken to make a choice during the probe stage. It was not possible to use litter as a factor in the cognitive bias analyses as less than three animals per litter were used in some groups. However, at least three litters were used per group, and visual inspection of the data demonstrated that data did not cluster by litter, and this can be seen in Figure 2.

## Results

### Bodyweight

Control animals were significantly heavier than JS animals (F_1,8.54_ = 8.76, *P* = 0.02), and this difference occurred from PND 42, two weeks after the administration of juvenile stress. Males were significantly heavier than females (F_1,759.4_ = 740.1, *P*<0.0001), and all animals gained weight as the weeks progressed (F_14,503.2_ = 655.9, *P*<0.0001). A significant sex*age interaction (F_14,753.3)_ = 30.83, *P*<0.0001), demonstrated that males were only heavier than females from PND 42 (Figure 3).

### EPM

JS animals spent significantly less time on the open arms of the EPM than control animals in both sexes (F_1,47_ = 10.64, *P* = 0.002) (Figure 4A).

### Cognitive Bias

JS animals made significantly more optimistic choices than control animals (F_1,22_ = 4.7, *P* = 0.04) (Figure 4B). Control animals made significantly less optimistic (or significantly more pessimistic) choices than expected by chance (Chi-squared test: X^2^ = 5.3, *P* = 0.02), and JS animals made chance levels of optimistic/pessimistic responses (Chi-squared test: X^2^ = 0.3, *P* = 0.56) (Figure 4B).

Females learnt the cognitive bias task significantly faster than males (F_1,22_ = 10.24, *P* = 0.004), and were less likely to make an incorrect choice during the training stage (F_1,24.97_ = 27.2, *P*<0.0001) (Figure 5A). Overall, control animals took longer to make a choice during this phase than JS animals (F_1,22_ = 4.43, *P* = 0.046), and all animals chose faster on chocolate compared to Cheerio trials (F_1,23_ = 13.40, *P* = 0.001) (Figure 5B).

Control animals took longer to make a decision during ambiguous probe compared to regular trials, and longer on ambiguous probe trials than JS animals took on both ambiguous probe and regular trials (group*trial type interaction: F_1,21_ = 9.83, *P* = 0.005, post-hoc Tukey HSD test) (Figure 6A). Females took longer on ambiguous than regular trials, and longer on ambiguous probe trials than males took on ambiguous probe and regular trials (trial type*sex interaction: F_1,21_ = 37.75, *P*<0.0001, post-hoc Tukey HSD test) (Figure 6B). There was no interaction between sex and group.

## Discussion

### Bodyweight

Animals that received juvenile stress were lighter than their control counterparts two weeks after the administration of stress (PND 42), and this difference persisted until PND 105. The same pattern has been found in previous juvenile stress studies: in [42] differences persisted until PND 68, and [43] found similar, but shorter term effects. Prenatal and early post natal stress often lead to long lasting decreases in bodyweight in animal models [44], [45], and underlying mechanisms are thought to include decreases in the secretion of essential enzymes for normal cell growth, a reduction in DNA synthesis, abnormal patterns of endocrine secretion, and a suppression of cell responses to growth hormone [46]. Further studies would have to be conducted to determine if the same mechanisms underlie juvenile stress induced reductions in bodyweight.

### EPM

The EPM was used to assess the effects of JS on anxiety-type behavior. Adults that had experienced JS displayed greater levels of anxiety-like behaviour, as they spent significantly less time than controls in the open arms of the EPM. This confirms what has been found in previous studies [11], [43], and has been specifically related to stress in the juvenile phase [9]. Furthermore, this reflects what is found in human populations, where childhood adversity is strongly associated with the development of anxiety disorders in adulthood [36].

### Cognitive Bias

Control animals responded in a pessimistic manner when presented with an ambiguous choice. This supports what has previously been found, and is discussed further in [31]. Animals that had received JS made significantly more optimistic choices than control animals. We predicted that early life stress would result in a more negative cognitive bias, as is observed in depressed and anxious human populations [20], [21]. A possible explanation for the relative increase in optimism found here is that stressed animals were more optimistic, or risk prone, about a high value reward because of their lower body weight. Animals that have been subjected to stress and experienced a subsequent decrease in body weight have been found to increase their risk taking whilst foraging, resulting in increased food intake and compensatory growth [47], [48]. It is possible that the JS rats were employing a similar strategy, and were therefore taking a greater risk for the higher-energy reward when presented with an ambiguous stimulus in the present study. These results may be specific to a foraging context, and it would be interesting to use a cognitive bias task that did not involve food, to determine the generalisability of these results. This result highlights the complications inherent in measuring emotional states in animal models of juvenile stress.

Closer inspection of the data revealed that control animals took longer to choose a reward bowl when presented with an ambiguous stimulus (intermediate grade sandpaper) compared to a stimulus they had been previously trained with (rough or smooth sandpaper), whereas JS animals did not. When an individual has to make a decision or choice between two alternatives, it will sample information from the environment until some threshold of neuronal activity is reached, and then a decision will be made [49]. As the difficulty of a choice task increases, so does neuronal activity, and this correlates with an increased time taken to make a decision [50], [51], [52]. One factor that increases the difficulty of a task and hence reaction time is the discriminability of presented stimuli [51], [53]. In the experiment described here, presenting the animals with an ambiguous stimulus increases the difficulty of the task, and so theoretically the time taken to make a decision. The ambiguous stimulus does not match either of the stimuli the animals have learned to associate with particular reward outcomes; its texture is in between that of both trained stimuli, making it harder to discriminate and match with confidence to either. Based on theory and previous empirical evidence [51], [53], we would predict that animals would sample this ambiguous information for longer, and therefore take longer to make a decision in this situation. Indeed, this is what we find with control animals. However, this process is altered in JS animals, as they make a choice at the same speed in trials with learned and ambiguous stimuli. One interpretation of this is that JS animals are acting impulsively when presented with an ambiguous stimulus. Altered decision making is found in humans with a range of neuropsychiatric disorders, including trait anxiety [54], major depression [55] and in suicidal individuals [56], and adults that have experienced childhood stress are at greater risk of developing impulse control disorders [57].

When presented with an ambiguous stimulus, females from both groups took longer than males to make a choice. Interestingly, there is evidence in the human literature that women are more averse to risk and ambiguity when making decisions [58]. We also found that females learned the cognitive bias task significantly faster than males. The literature on sex differences in learning in rodent models is not straightforward: most studies find no difference, some a male and some a female advantage [59]. It is not clear why females learned the task presented here faster than males.

## Conclusion

In conclusion, we found that juvenile stress increases anxiety-like behaviour and decreases decision-making time to ambiguous cues in a rat model. This reflects the affective disturbances and impaired decision making seen in human clinical disorders related to childhood adversity. Future work should be directed at investigating the underlying mechanisms of such behavioural changes, and at assessing potential therapeutic interventions (e.g. environmental enrichment, pharmacological administration), with the aim of improving human medicine.

## References

[1] Anda RF, Felitti VJ, Bremner JD, Walker JD, Whitfield C, et al (2006) The enduring effects of abuse and related adverse experiences in childhood – A convergence of evidence from neurobiology and epidemiology. Eur Arch Psychiatry Clin Neurosci 256: 174–186. 16311898 10.1007/s00406-005-0624-4 PMC3232061

[2] Heim C, Newport DJ, Mletzko T, Miller AH & Hemeroff (2008) CB (2008) The link between childhood trauma and depression: Insights from HPA axis studies in humans. Psychoneuroendocrinology 33: 693–710. 18602762 10.1016/j.psyneuen.2008.03.008

[3] Bale TL, Baram TZ, Brown AS, Goldstein JM, Insel TR, et al (2010) Early Life Programming and Neurodevelopmental Disorders. Biol Psychiatry 68: 314–319. 20674602 10.1016/j.biopsych.2010.05.028 PMC3168778

[4] Pechtel P, Pizzagalli DA (2011) Effects of early life stress on cognitive and affective function: an integrated review of human literature. Psychopharmacol 214: 55–70. 10.1007/s00213-010-2009-2 PMC3050094 20865251

[5] Romeo RD, McEwen BS (2006) Stress and the adolescent brain. Resilience in Children 1094: 202–214. 10.1196/annals.1376.022 17347352

[6] Morgan L, Scourfield J, Williams D, Jasper A, Lewis G (2003) The Aberfan disaster: 33-year follow-up of survivors. Br J Psychiatry 182: 532–536. 12777345 10.1192/bjp.182.6.532

[7] Kausch O, Rugle L, Rowland DY (2006) Lifetime histories of trauma among pathological gamblers. Am J Addict 15: 35–43. 16449091 10.1080/10550490500419045

[8] Weich S, Patterson J, Shaw R, Stewart-Brown S (2009) Family relationships in childhood and common psychiatric disorders in later life: systematic review of prospective studies. Br J Psychiatry 194: 392–398. 19407266 10.1192/bjp.bp.107.042515

[9] Avital A, Richter-Levin G (2005) Exposure to juvenile stress exacerbates the behavioural consequences of exposure to stress in the adult rat. Int J Neuropsychopharmacol 8: 163–173. 15546500 10.1017/S1461145704004808

[10] Toledo-Rodriguez M, Sandi C (2007) Stress before puberty exerts a sex- and age-related impact on auditory and contextual fear conditioning in the rat. Neural plast 71203: 1–12. 10.1155/2007/71203 PMC1931496 17671613

[11] Tsoory M, Cohen H, Richter-Levin G (2007) Juvenile stress induces a predisposition to either anxiety or depressive-like symptoms following stress in adulthood. Eur Neuropsychopharmacol 17: 245–256. 16889944 10.1016/j.euroneuro.2006.06.007

[12] Jacobson-Pick S, Richter-Levin G (2010) Differential impact of juvenile stress and corticosterone in juvenility and in adulthood in male and female rats. Behav Brain Res 214: 268–276. 20561965 10.1016/j.bbr.2010.05.036

[13] Jacobson-Pick S, Elkobi A, Vander S, Rosenblum K, Richter-Levin G (2008) Juvenile stress-induced alteration of maturation of the GABA(A) receptor alpha subunit in the rat. Int J Neuropsychopharmacol 11: 891–903. 18364065 10.1017/S1461145708008559

[14] Tsoory MM, Guterman A, Richter-Levin G (2010) “Juvenile Stress” alters maturation-related changes in expression of the neural cell adhesion molecule L1 in the limbic system: relevance for stress-related psychopathologies. J Neurosci Res 88: 369–380. 19746433 10.1002/jnr.22203

[15] Barha CK, Brummelte S, Lieblich SE, Galea LAM (2011) Chronic restraint stress in adolescence differentially influences hypothalamic-pituitary-adrenal axis function and adult hippocampal neurogenesis in male and female rats. Hippocampus 21: 1216–1227. 20665592 10.1002/hipo.20829

[16] Eiland L, Ramroop J, Hill MN, Manley J, McEwen BS (2012) Chronic juvenile stress produces corticolimbic dendritic architectural remodeling and modulates emotional behavior in male and female rats. Psychoneuroendocrinology 37: 39–47. 21658845 10.1016/j.psyneuen.2011.04.015 PMC3181388

[17] Tsoory M, Richter-Levin G (2006) Learning under stress in the adult rat is differentially affected by ‘juvenile’ or ‘adolescent’ stress. Int J Neuropsychopharmacol 9: 713–728. 16321169 10.1017/S1461145705006255

[18] Mathews A, MacLeod C (2005) Cognitive vulnerability to emotional disorders. Annu Rev Clin Psychol 1: 167–195. 17716086 10.1146/annurev.clinpsy.1.102803.143916

[19] Koster EHW, Fox E, MacLeod C (2009) Introduction to the Special Section on Cognitive Bias Modification in Emotional Disorders. J Abnorm Psychol 118: 1–4. 19222308 10.1037/a0014379

[20] Wells A, Matthews G (1996) Anxiety and cognition. Curr Opin Psychiatry 9: 422–426.

[21] Amir N, Beard C, Bower E (2005) Interpretation bias and social anxiety. Cognit Ther Res 29: 433–443. 10.1007/s10608-009-9258-6 PMC2872495 20495620

[22] Rude SS, Valdez CR, Odom S, Ebrahimi A (2003) Negative cognitive biases predict subsequent depression. Cognit Ther Res 27: 415–429.

[23] Rude SS, Durham-Fowler JA, Baum ES, Rooney SB, Maestas KL (2010) Self-report and cognitive processing measures of depressive thinking predict subsequent major depressive disorder. Cognit Ther Res 34: 107–115.

[24] Paul ES, Harding EJ, Mendl M (2005) Measuring emotional processes in animals: the utility of a cognitive approach. Neurosci Biobehav Rev 29: 469–491. 15820551 10.1016/j.neubiorev.2005.01.002

[25] Brydges NM, Braithwaite VA (2008) Measuring Animal Welfare: What Can Cognition Contribute? ARBS Ann Rev Biomed Sci 10: T91–T103.

[26] Mendl M, Burman OHP, Parker RMA, Paul ES (2009) Cognitive bias as an indicator of animal emotion and welfare: Emerging evidence and underlying mechanisms. Appl Anim Behav Sci 118: 161–181.

[27] Harding EJ, Paul ES, Mendl M (2004) Animal behavior – Cognitive bias and affective state. Nature 427: 312–312. 14737158 10.1038/427312a

[28] Burman OHP, Parker RMA, Paul ES, Mendl MT (2009) Anxiety-induced cognitive bias in non-human animals. Physiol Behav 98: 345–350. 19560479 10.1016/j.physbeh.2009.06.012

[29] Bateson M, Matheson SM (2007) Performance on a categorisation task suggests that removal of environmental enrichment induces ‘pessimism’ in captive European starlings (Sturnus vulgaris). Anim Welf 16: 33–36.

[30] Bateson M, Desire S, Gartside SE, Wright GA (2011) Agitated honeybees exhibit pessimistic cognitive biases. Curr Biol 21: 1070–1073. 21636277 10.1016/j.cub.2011.05.017 PMC3158593

[31] Brydges NM, Leach M, Nicol K, Wright R, Bateson M (2011) Environmental enrichment induces optimistic cognitive bias in rats. Anim Behav 81: 169–175.

[32] Enkel T, Gholizadeh D, von Bohlen und Halbach O, Sanchis-Segura C, Hurlemann R, et al (2010) Ambiguous-Cue Interpretation is Biased Under Stress- and Depression-Like States in Rats. Neuropsychopharmacol 35: 1008–1015. 10.1038/npp.2009.204 PMC3055368 20043002

[33] Mendl M, Brooks J, Basse C, Burman O, Paul E, Blackwell E, et al (2010) Dogs showing separation-related behaviour exhibit a ‘pessimistic’ cognitive bias. Curr Biol 20: R839–R840. 20937467 10.1016/j.cub.2010.08.030

[34] Salmeto AL, Hymel KA, Carpenter EC, Brilot BO, Bateson M, et al (2011) Cognitive bias in the chick anxiety-depression model. Brain Res 1373: 124–130. 21156165 10.1016/j.brainres.2010.12.007

[35] Hymel KA, Sufka KJ (2012) Pharmacological reversal of cognitive bias in the chick anxiety-depression model. Neuropharmacol 62: 161–166. 10.1016/j.neuropharm.2011.06.009 21722654

[36] Green JG, McLaughlin KA, Berglund PA, Gruber MJ, Sampson NA, et al (2010) Childhood adversities and adult psychiatric disorders in the national comorbidity survey replication I: associations with first onset of DSM-IV disorders. Arch Gen Psychiatry 67: 113–12. 20124111 10.1001/archgenpsychiatry.2009.186 PMC2822662

[37] Mueller BR, Bale TL (2008) Sex-specific programming of offspring emotionality after stress early in pregnancy. J Neurosci 28: 9055–9065. 18768700 10.1523/JNEUROSCI.1424-08.2008 PMC2731562

[38] Goel N, Bale TL (2009) Examining the intersection of sex and stress in modelling neuropsychiatric disorders. J Neuroendocrinol 21: 415–420. 19187468 10.1111/j.1365-2826.2009.01843.x PMC2716060

[39] Bao A-M, Swaab DF (2010) Sex differences in the brain, behavior and neuropsychiatric disorders. Neuroscientist 16: 550–565. 20889965 10.1177/1073858410377005

[40] Vigues S, Dotson CD, Munger SD (2009) The receptor basis of sweet taste in mammals. Chemosensory Systems in Mammals Fishes and Insects 47: 187–202. 10.1007/400_2008_2 19083128

[41] Box GEP, Cox DR (1964) An analysis of transformations. J R Stat Soc Series B Stat Methodol 26: 211–252.

[42] Yee N, Ribic A, de Roo CC, Fuchs E (2011) Differential effects of maternal immune activation and juvenile stress on anxiety-like behaviour and physiology in adult rats: No evidence for the “double-hit hypothesis”. Behav Brain Res 224: 180–188. 21679729 10.1016/j.bbr.2011.05.040

[43] Ilin Y, Richter-Levin G (2009) Enriched environment experience overcomes learning deficits and depressive-like behavior induced by juvenile stress. Plos One 4 10.1371/journal.pone.0004329 PMC2631645 19180243

[44] Chapillon P, Patin V, Roy V, Vincent A, Caston J (2002) Effects of pre- and postnatal stimulation on developmental emotional and cognitive aspects in rodents: A review. Dev Psychobiol 41: 373–387. 12430161 10.1002/dev.10066

[45] Viveros M-P, Llorente R, Diaz F, Romero-Zerbo SY, Bermudez-Silva FJ, et al (2010) Maternal deprivation has sexually dimorphic long-term effects on hypothalamic cell-turnover body weight and circulating hormone levels. Horm Behav 58: 808–819. 20708008 10.1016/j.yhbeh.2010.08.003

[46] Kuhn CM, Schanberg SM (1998) Responses to maternal separation: Mechanisms and mediators. Int J Dev Neurosci 16: 261–270. 9785122 10.1016/s0736-5748(98)00034-3

[47] Killen SS, Marras S, McKenzie DJ (2011) Fuel fasting fear: routine metabolic rate and food deprivation exert synergistic effects on risk-taking in juvenile European sea bass. J Anim Ecol 80: 1024–1033. 21790592 10.1111/j.1365-2656.2011.01844.x

[48] Damsgard B, Dill LM (1998) Risk-taking behaviour in weight-compensating coho salmon Oncorhynchus kisutch. Behav Ecol 9: 26–32.

[49] Gold JI, Shadlen MN (2007) The neural basis of decision making. Annu Rev Neurosci 30: 535–574. 17600525 10.1146/annurev.neuro.29.051605.113038

[50] Gould RL, Brown RG, Owen AM, Ffytche DH, Howard RJ (2003) fMRI BOLD response to increasing task difficulty during successful paired associates learning. Neuroimage 20: 1006–1019. 14568470 10.1016/S1053-8119(03)00365-3

[51] Smith PL, Ratcliff R (2004) Psychology and neurobiology of simple decisions. Trends Neurosci 27: 161–168. 15036882 10.1016/j.tins.2004.01.006

[52] Heekeren HR, Marrett S, Ungerleider LG (2008) The neural systems that mediate human perceptual decision making. Nat Rev Neurosci 9: 467–479. 18464792 10.1038/nrn2374

[53] Pleskac TJ, Busemeyer JR (2011) “Two-stage dynamic signal detection: A theory of choice decision time and confidence”. Erratum Psychol Rev 118: 56. 10.1037/a0019737 20658856

[54] Miu AC, Heilman RM, Houser D (2008) Anxiety impairs decision-making: psychophysiological evidence from an Iowa Gambling task. Biol Psychiatry 77: 353–358. 10.1016/j.biopsycho.2007.11.010 18191013

[55] Cella M, Dymond S, Cooper A (2010) Impaired flexible decision-making in major depressive disorder. J Affective Disorder 124: 207–210. 10.1016/j.jad.2009.11.013 20004023

[56] Jollant F, Bellivier F, Leboyer M, Astruc B, Torres S, et al (2005) Impaired decision making in suicide attempters. Am J Psychiatry 162: 304–310. 15677595 10.1176/appi.ajp.162.2.304

[57] Brosdky BS, Oquendo M, Ellis SP, Haas GL, Malone KM, et al (2001) The relationship of childhood abuse to impulsivity and suicidal behavior in adults with major depression. Am J Psychiatry 158: 1871–1877. 11691694 10.1176/appi.ajp.158.11.1871

[58] Charness G, Gneezy U (2012) Strong evidence for gender differences in risk taking. J Econ Behav Organ 83: 50–58.

[59] Jonasson Z (2005) Meta-analysis of sex differences in rodent models of learning and memory: a review of behavioral and biological data. Neurosci Biobehav Rev 28: 811–825. 15642623 10.1016/j.neubiorev.2004.10.006

